# Reviews

**Published:** 1896-12

**Authors:** 


### REVIEWS.

NOVEMBER *Veterinarian.* Telegony experiments by Professor Ewart. Address of Professor Finlay Dun at the opening of the Dick Veterinary College, Edinburgh. Some points in the evolution of the horse. Gastric tympany treated with terebene in from two- to four-ounce doses, with chlorodyne in about same quantities, sweet spirits of nitre, two ounces, combined with a pint and a half of linseed oil. An outbreak of hoose, or husk, in calves is reported; treatment by intra-tracheal injections of carbolic acid, ♏v; chloroform, ♏xv; oil of turpentine and glycerin in equal parts, one drachm, did not prove very effective. Three cases of dropped elbow are reported, all of which made prompt recoveries; and, in summing up the treatment, Veterinarian Brown advises a shoe with an elongated toe-piece, to flatten out the projection, and through the flattened part bore three or four holes; the toe-piece should then be turned up so as to assume a horizontal position; then make a splint, which should have its lower end planed off in a slanting direction, so that, with the flat surface toward the anterior surface of the foot, it (the end of the splint) is inserted between the foot and the toe-piece; screws should then fix it in that position. The splint should extend up to within a few inches of the elbow-joint, and should be firmly padded, so that a fit and proper pressure may be maintained. Straps, three in number—one for the metacarpal region, one for the knee, and the other above the knee—about two inches broad, should be nailed to the anterior surface of the splint and firmly bound, with the aid of tow as padding, around the limb. All that remains to be done is, if a heavy animal, to supply slings for support, fomentation in the early stages, stimulating embrocation later, and, finally, if necessary, a good strong blister. A new mouth-gag for dogs and cats. The value of Röntgen rays in veterinary diagnosis for splint or fractured pastern, fractured coffin-bone; the presence, or otherwise, of side-bone, ring-bone, and spavin; the extent of punctured wounds of foot, fractures of the ribs, the vertebræ, and the presence and location of intestinal calculi. The Southern Counties Veterinary Medical Association is agitating measures for exempting from jury duty, revision of fees now paid when giving evidence in criminal cases to the same basis as solicitors and medical men. President James McI. McCall, after reviewing the methods and use of mallein for the diagnosis of glanders, presented the following proposition before the West of Scotland Veterinary Medical Association, recognizing the value of mallein as a diagnostic agent in suspected glanders, recommends that the opinion of the various veterinary medical associations be sought upon this subject, in order to decide the advisability of memorializing the Council of the Royal College of Veterinary Surgeons to lay before the Board of Agriculture the following suggestion: "When glanders occurs in a stud of horses compulsory testing with mallein should be performed upon each animal in it, and each horse which responds to the test should be thereafter slaughtered and compensation paid to the owner out of the funds of the Board." Prof. McCall further said that he felt justified in coming to the conclusion that as a test for glanders mallein may be considered almost infallible.

*The Horseshoer and Hardware Journal* has now become the official organ of the National Master Horseshoers' Protective Association; it will be published in Detroit under the general direction of Mr. J. C. Buckley, ex-President of the National Association. Drs. S. K. Johnson and H. D. Gill come in for a generous round of praise at the fifth annual convention of Master Shoers in New York City, for their kindly interest in the success of the establishment of a law and board of examiners in the interest of advancement among the craft. Veterinarians Robertson, of Chicago; McKenzie, of Rochester; McAnulty, of Philadelphia; and Quinn, of New York, were actively interested in the National Shoers' Convention. Chairman Robertson reported in appreciative words the results of their visit to the Buffalo Convention of the U. S. V. M. A., and the warm friends they met there. He also announced in his report the early publication of two works o special import and value to the shoeing craft, one by Prof. Adams of the Veterinary Department of the University of Pennsylvania; a second one by Prof. Hughes of the Chicago Veterinary College, bearing upon the subject of anatomy and such features of anatomical study of import to students of the art of farriery. The kindliest feeling toward the veterinary profession was a marked feature of the convention, and for aid, direction, and support they realized that it was well to rest upon such an arm of support. Dr. James T. McAnulty, of

Philadelphia, presented a paper on "The Horseshoer and the Veterinarian, their Relation to Each Other." He opened his paper by saying the profession of veterinary science and farriery, though in practice should be entirely distinct, have, nevertheless, a mutual dependence so complete that the excellence and usefulness of the one are materially affected by any deficiency in the other. He advised all farriers to abstain from treating diseases of the feet. Addressing directly his fellow-craftsmen, he said: "You regard with little respect the individual who empirically practises your own vocation, how must the educated veterinarian regard the farrier who, without suitable preparation, attempts to practise veterinary medicine?"

The *American Veterinary Review* for November. W. H. Lawes records a case of recovery from tetanus, under drachm doses of cannabis indica and thirty-grain doses of bromide of potassium. Poisoning by loco-weed: twenty-three deaths in a year out of a bunch of three hundred horses; change of pasture proved of no value to the sick. A case of missed labor, malpresentation, Cæsarean section in a cow, recovery complete; calf weighed over one hundred pounds and stretched out over six feet in length; tar was used as an external dressing to the incision in the flank; sweet spirits of nitre and acetanilid were used as internal treatment.

The *Veterinarian* for October. Professor Dewar, on the "Influence of Heredity in Disease," traces up the tendency to splints, curbs, spavins, side-bones, and windgalls as abnormal conditions that follow strongly in certain families, and lays great stress on his final conclusion that an organism tends to develop in the likeness of its progenitors. A case of metastatic laminitis following metritis is recorded. The discovery of iodine as a constituent of the animal body, particularly of certain glandular structures, and its value in reducing these growths when diseased, are treated of.

The *Journal of Comparative Pathology and Therapeutics* for the quarter ending with September. Dr. Frederick Hobday reports 36 cases operated on for various diseased lesions below the knee, by median neurectomy; of this number 7 remain under treatment, 2 were in no way improved, and 34 have been sent to work; 18 are known to be working regularly, 2 have since died from other causes, 1 worked for ten weeks and was then sold and was lost sight of, and 3 were untraced; 5 animals were operated upon in both fore-legs. Professor Hobday further says for old-standing lameness, where due to splints, exostoses anywhere on the inside of the leg, chronically sprained, thickened and painful tendons, or cases of that kind which cause pain by pressing on the adjacent nerve-structures, after all other treatment has failed, the operation is certainly to be recommended; but for ringbones, side-bones, exostoses on the fetlock, navicular disease, or lameness caused by anything below the usual seat of plantar neurectomy, there is no advantage over this last operation; in fact, there is the disadvantage that one has unnecessarily deprived a large portion of the leg of its normal nerve-supply. It is worthy of notice that although in some cases there was immediate improvement when the animal was trotted after the operation, in others this was very slight, but as the wound healed the lameness completely disappeared. Professor J. McFadyean reports in detail the history and autopsical lesions of five cases of equine tuberculosis. In conclusion, he thought it might be justifiably held that there is still strong reason to suspect cow's milk as the most common vehicle of infection in tuberculosis of the horse, and that the practice of prescribing raw cow's milk for sick horses, or of giving it as a fattening article of diet to stallions, is attended with a considerable degree of risk unless the cows that furnish the milk are known to be free from tuberculosis. A case of scirrhous cord is reported which was successfully operated upon by surgical extirpation, from which microscopical sections were made in which were found botryomycosis equi (Bollinger). Professor A. Sheridan Delépine's address before the British Medical Association "On the Place of Pathology in Medical Education." A strong editorial showing the unreliable character of the claims of Professor Arloing as to the discovery of the cause of the disease contagious pleuro-pneumonia, and a comparison of the tests with his prepared lymph for inoculation and that of Willem's method. The latter fully justified the claims and former experiences, while the former failed to either produce curative or protective influences, and therefore adds a danger of false security where it is used.

---

Dr. G. A. Johnson, of Sioux City, Iowa, reports absolute failure in the treatment of a case of tetanus with fluid-extract of gelsemium.

